# The Uræmic Convulsions of Pregnancy, Parturition, and Childbed

**Published:** 1858-11

**Authors:** 


ART. III.—*The Uræmic Convulsions of Pregnancy, Parturition, and Childbed.* By DR. CARL R. BRAUN, Professor of Midwifery, Vienna. Translated from the German, with Notes, by J. MATTHEWS DUNCAN, F.R.C.P.E., Lecturer on Midwifery, etc. 12mo., pp. 182. New York: S. S. and W. Wood, 1858.

THIS little volume constitutes a single chapter in Dr. Braun's recent work on obstetrics, published at Vienna in 1857, and entitled *Lehrbuch der Geburtshülfe mit Einschluss der operativen Therapeutik, der übrigen Fortpflanzungs-functionen der Frauen und der Puerperalprocesse.* Dr. Duncan informs us in his preface that this chapter appeared to him so valuable, from its completeness and erudition, that he was induced to publish a translation of it in the *Edinburgh Medical Journal* for 1856–57. Partly in consequence of the favorable manner in which it was received by the readers of that journal, and partly from the want of any similar treatise in the English language, it has been republished in Edinburgh in its present separate form, of which the copy before us is the American reprint.

The work is divided into ten chapters, of which the first two are devoted to an account of the symptoms and pathogeny of the disease.

Eclampsia puerperalis is defined by Dr. Braun to be "an acute affection of the motor function of the nervous system (an acute neurosis of motility) characterized by insensibility, tonic, and clonic spasms, and occurs only as an accessory phenomenon of another disease, generally of Bright's disease in an acute form, which, under certain circumstances, spreading its toxæmic effects on the nutrition of the brain and whole nervous system, produces those fearful accidents." Unfortunately the disease has an extensive synonymy. It is the epilepsia ex utero of the ancients, the dystocia convulsiva of Young, the dystocia epileptica of Merriman, the encephalopathia albuminurica of Legroux, and the allgemeine Krämpfe, or schwere Convulsionen of Wigand. In this country and in Great Britain it is called by different writers puerperal convulsions, acute epilepsy, renal spasms, uræmic convulsions, epilepsia renalis, albuminurica, eclampsia gravidarum, parturientium et puerperarum. With the French it is the convulsions des femmes enceintes et en couche.

Under the general name of "Eclampsia" have been confounded several pathological conditions differing from each other in many of their symptoms, though exhibiting in common both tonic and clonic spasms, and accompanied with loss of sensibility and great danger to the life of the patient. The eclampsia gravidarum, according to Dr. Braun, is commonly due to that form of blood-poisoning which results from the retention and decomposition of urea in the blood, or the retention of the excrementitial, extractive matter of the urine. Hence uræmic eclampsia is not peculiar to pregnancy, since toxæmic conditions may give rise to it not only in the non-pregnant female, but also in children and in men. Another form of eclampsia, presenting somewhat different symptoms, results from the defective elimination of carbonic acid, bile and other matters which in a state of health are freely and constantly separated from the blood. There is also a phlegmasial variety of eclampsia, known as the cerebral or apoplectic, and originating from meningitis, encephalitis, intermeningeal apoplexy, thrombosis of the longitudinal sinuses, and hyperæmia of the brain, spinal cord or medulla oblongata. Eclamptic attacks are sometimes very closely simulated by hysterical convulsions resulting from the reflection of some peripheral irritation upon the medulla spinalis or oblongata. Chemical and histological alterations of the serum and red corpuscles of the blood, as in hydræmia, leukæmia, hyperinosis, etc., are capable of producing this disease. Death following anæmia, brought on by a sudden and copious loss of blood, is often accompanied with eclamptic phenomena. Conditions similar to eclampsia are occasionally produced by mineral, animal, and vegetable poisons, such as preparations of lead, strychnia, conium maculatum, cicuta aquatica, œnanthe crocata, etc., and the inhalation of carbonic acid and carbonic oxide gases.

This formidable disease is characterized by a series of convulsive paroxysms rapidly repeated and accompanied with a complete loss of sensibility which is prolonged into the intervals between the fits. During a paroxysm the face and neck are swollen, injected, and of a bluish color; the carotids pulsate forcibly; the eyelids are prominent; the eyeballs roll convulsively in different directions; the capillaries of the conjunctiva are injected; the veins of the neck and face are turgid; the facial muscles strongly distorted; the mouth wide open; and the tongue generally protruded and often bitten; the trunk is twisted to one side, and all the extremities thrown into jerking motions. The respiratory muscles, especially the diaphragm, are in a state of contraction, so that respiration is seriously interfered with, and even asphyxia sometimes produced. Involuntary discharges of urine and fæces take place. Occasionally vomiting precedes the first fit. The skin is sometimes dry and hot, sometimes cool and moist. In some cases the pulse is frequent, in others slow. These symptoms are followed by a soporose condition in which the patient lies motionless, devoid of consciousness and sensation, and with the extremities stretched out and stiff. In this state the respiration is at first hurried, difficult and stertorous, afterwards slow and snoring. Sometimes, though very rarely, this sopor deepens into coma and lasts a whole day. Generally, however, after a period of time, varying from one-half to one or two hours, the paroxysm, if not fatal, remits; the respiration becomes slower and freer; muscular rigidity diminishes; the pulse is less frequent; the abdomen tender to the touch, consciousness returns slowly and very slightly, if at all, while the reflex sensibility, which during the fit was suspended, is often intense.

"After awaking, patients generally complain of a confused, dull headache, and of great languor, which continue till a renewal of restlessness, stretching, extending, slow tremulous bending of the upper extremities, jerking of the facial muscles, with reddening of the face, announce a new paroxysm. The fits may be repeated several times in a day—sometimes as much as seventy times in a day. Generally after a few fits, complete unconsciousness supervenes, and this continues till recovery or death. Sometimes there are phenomena foreboding the coming on of fits. They consist of headache, giddiness, wandering of the ideas, increased heat of skin, mental illusions, seeing of lights, imperfectness of vision even to total blindness, (amblyopia, amaurosis,) with an enlarged pupil and staring look, noises in the ears, difficulty of speaking, lowness of spirits, pains in the præcordium, nausea, vomiting, palsies, irregular pulse, and great languor without any known cause."

It is well known that œdema, amounting sometimes to anasarca, often precedes and accompanies eclampsia gravidarum. This œdema, according to Prof. C. D. Meigs, is due to the same cause which produces the eclampsic attack also—the pressure of the gravid uterus upon the great arterial trunk and its main branches, and upon the veins and absorbent vessels in the lumbar region. Local or general infiltration of the areolar tissue, and over-distension of the encephalic blood-vessels are in his opinion cotemporaneous results of this pressure. Hence he regards this œdema, especially if it be accompanied with headache, tinnitus aurium, muscæ volitantes, vertigo, etc., as a significant warning of an approaching convulsion, and accordingly resorts to venesection ad deliquium to arrest the disease. Dr. Braun, on the other hand, after analyzing the numerous views entertained as to the cause of eclampsia, arrives at the conclusion that "the eclampsic convulsions of women during pregnancy must be considered to be identical with the fits of adults, in general, that are produced by uræmia in the course of acute Bright's disease." This doctrine, which he regards as "an axiom in theory as well as in practice," he announced in 1851, about the same time that Frerichs published his well-known essay on this subject. Concerning the concomitant œdema, he says:—

"Only those œdemata of pregnant women which exist contemporaneously with albumen, fibrin cylinders, and fatty degenerated scales of Bellini's epithelium in the urine, have a connection with uræmic eclampsia. The œdema of the lower extremities, ascites, and hydramnios, which are not complicated with albuminous urine containing fibrin cylinders, are not followed by uræmic eclampsia in pregnancy or labor. The affection of the kidneys with disease cannot with certainty be inferred from the appearance of dropsy, as distinct causes may, at the same time, or one after the other, produce dropsies."

The coincidence of eclampsia and albuminuria has been frequently verified by Lever, Simpson, Devilliers, Regnault, Dubois, Danyau, Cazeaux, Cormack, Blot, Helfft, Frerichs, Litzmann, Braun, and many others. These two conditions, however, are not necessarily related to each other as cause and effect, for abundant observation has shown that in the progress of many diseases albumen may appear in the urine without the development of uræmic intoxication and convulsions. Albuminuria has been detected in persons laboring under articular rheumatism, thoracic inflammation, intermittent and typhus fevers, measles, cholera, chronic hepatic affections, etc. According to Robin, "the urine becomes albuminous in croup, in ascites, and in cases of capillary bronchitis, with emphysema, accompanied by dyspnœa; in pulmonary phthisis, in gestation when sufficiently advanced to occasion an habitual congestion of the kidneys; in cyanosis, diabetes, etc." Simon found albumen in the urine of persons apparently in good health; while Tegart, thirteen years ago, and Hammond, quite recently, experimenting upon themselves, detected albumen in their urine after feeding freely upon the white of egg. It is now pretty well settled that albuminous urine may result either from some alteration in the composition of the blood, from pressure upon the renal veins, or from some structural or functional change in the kidneys, and that it may exist independently of uræmia and puerperal convulsions. In fact albuminuria, general or local œdema, and puerperal convulsions, often coexist without being directly related to, or dependent upon each other.

Again, dropsy may occur during pregnancy, and the urine remain in a normal condition. In cases of albuminuria associated with œdema and eclampsia, our author assures us that the quantity of albumen has generally an intimate relation to the extent, intensity, and duration of the acute Bright's disease, which he regards as the primary cause of eclampsia, and not so constantly to the violence of the latter affection. The albumen generally increases, however, at the end of pregnancy, during labor, and during the eclampsic fits. In 1847, Cahen, in a thesis entitled "*De la Néphrite albumineuse chez les Femmes enceintes*," attempted to prove that the albuminuria of gestation could in all cases be traced to disease of the kidney. M. Blot subsequently opposed this view. He detected disease of the kidney in three only of seven women who died while laboring under albuminuria. It is well known that in almost every case albumen rapidly disappears from the urine, sometimes in a few hours after delivery. These facts, together with the absence of the characteristic renal symptoms, the greater abundance of the salts, particularly the urates, in the urine, and the consequent increase in the density of that fluid,—characters so different from those presented by the urine in Bright's disease,—emphatically negative the opinion of Cahen. It is probably true that by far the greater number of cases of œdema and albuminuria gravidarum are due to simple congestion of the kidney, resulting from the impeded renal circulation produced by pressure of the gravid uterus. It is equally probable also, that this pressure is the most common cause of œdema. But that eclampsia is always or even often produced by uterine pressure is by no means borne out by the facts.

"The influence of simple pressure by the pregnant uterus," says Dr. Duncan, "seems to be much exaggerated. For in cases of ovarian dropsy, fibrous tumor, and a variety of other affections, no such result is observed from a pressure exactly like that of the gravid uterus, and often more severe and longer continued. Moreover, it is well known that a great number of characteristic cases occur before the uterus has so increased in size as to cause much pressure, and many after all extraordinary pressure is removed by complete delivery."

The question here presents itself as to the cause of puerperal eclampsia. The recorded observations and experiments of Prevost and Dumas, Segalas, Tiedemann, Gmelin, Mitscherlich, Bernard, Barreswill, Bichat, Courten, Gaspard, Vanquelin, Stannius, Frerichs, Bright, Christison, Rees, and quite recently of Hammond, show pretty conclusively that urea may exist in abundance in the blood without giving rise to convulsions or death. In 1851, Frerichs was led by his experiments to attribute uræmic intoxication not to urea nor any other ingredient of the urine, but to the presence of carbonate of ammonia in the blood, which he supposes to be formed from the urea by the action of some ferment. He assures us that he detected this salt in the blood and exhalations in all cases in which the symptoms of uræmia were developed. Dr. Braun adopts without reserve this theory of Frerichs as a satisfactory explanation of the origin of eclampsia puerperalis. The very recent experiments of Dr. Hammond "On the Injection of Urea and other substances into the Blood," are decidedly opposed to the views advanced by Frerichs. Dr. Hammond was unable to discover ammonia either in the breath, vomited matter, or contents of the stomach of the animals into whose circulation he had injected urea, both alone and combined with vesical mucus. He thinks that the ammonia discovered by Frerichs in his experiments was of accidental occurrence, and is not to be regarded as an invariable attendant upon the retention of urea in the system. He found that the action of substances introduced into the blood is very much influenced by the presence or absence of the kidneys. Thus urea, carbonate of ammonia and sulphate of potash, injected into the blood-vessels of sound animals, do not cause death. Nitrate of potash thus introduced is speedily fatal. Convulsions and death ensue from the injection of any of the above-named substances into the circulation of animals whose kidneys have been previously extirpated. Whether the kidneys be present or absent, urea introduced directly into the blood undergoes no conversion into carbonate of ammonia.

"The condition of system," says Dr. Hammond, "remaining after extirpation of the kidneys, is, in many respects, analogous to that present during Bright's disease. In the latter condition the kidneys act imperfectly, and many substances which should be eliminated are retained in the organism. In the former there is, of course, no excretion of matter through these channels. In all cases, so far as I am aware, where the kidneys of animals have been extirpated and urea injected into the blood, death has supervened in a much shorter time than would have been the case had no urea been thus introduced into the system. I see, therefore, no great difficulty in ascribing the condition known as uræmic intoxication to the direct action of an excessive accumulation of urea in the system.

"The fact that urea in large quantity has been found in the blood of persons suffering under Bright's disease, but in whom no symptoms of blood-poisoning were present, is no argument against the correctness of this view; for, doubtless, as with most other poisons, all persons are not alike sensitive to its action. Moreover, when the disease progresses slowly the rate of accumulation of urea is also slow, and thus the system, by becoming in a manner habituated to its presence, may be enabled to endure an excess without symptoms of intoxication necessarily attending."*

It thus appears, from these conflicting statements, that the pathogenesis of this important disease is yet far from being satisfactorily established.

---

* See the number of this Journal for March, 1858.

The third and fourth chapters of the work before us treat of the connection of labor-pains with eclampsia, and the influence of the latter upon the life of the fœtus.

Dr. Braun cites numerous observers to show, as a matter beyond doubt, that eclampsia may appear independently of uterine pains.

"Even with the most violent eclampsia, sometimes no pains are observed; the fits may cease and pregnancy go on for weeks; or pains already become active may disappear, and days may pass ere parturition takes place spontaneously; or, during the continuance of the eclampsia, complete absence of pains may be remarked, the patient may die of the fits before labor is ended, and a dead fœtus be extracted by cæsarian section from the corpse."

Our author thinks that the pains should be regarded as the effect rather than the cause of eclampsia; and he asserts positively that fits cannot be produced at will, nor even aggravated by exciting pains and increasing their strength.

"We have made the observation," he says, "that under a high degree of reflex sensibility, convulsions cannot be induced at will at definite periods by violent irritation of the uterus; and that by the energetic use of the caoutchouc bladder-plug, very strong pains may indeed be produced, but no increased intensity, and no more frequent returns of the eclamptic fits, and that during colpeurysis* the convulsions themselves sometimes altogether cease, and the birth of the child takes place rapidly. * * * * The frequent occurrence of eclampsia during the first days of childbed evidently proves that there is a very subordinate relation between active pains and uræmic eclampsia. * * * * When the paroxysms are numerous, sometimes the appearance of a fit is contemporaneous with the acme of pain, sometimes with the interval between pains.

"Convulsions, when they appear suddenly, are probably produced by the sudden decomposition of a quantity of urea.

"Without uræmia, the most violent and painful uterine contractions produce no eclampsia in a case of Bright's disease; as, after long experience, we have frequently observed in women suffering from Bright's disease during pregnancy and natural labor.

"If it happens that the uræmia is removed during pregnancy or labor, then the later and stronger pains generally are followed by no more fits.

"With the disappearance of uræmia, the pains sometimes become weaker. During uræmia the uterus is never seized with tetanus. The consideration of these numerous facts makes it certain that the pains play a very subordinate part in cases of uræmic eclampsia.

---

* This is a method of inducing premature labor. It consists in distending the vagina by means of a colpeurynter, or bag of caoutchouc into which water is forcibly injected and there retained.

"Between eclampsia and the serous effusions there does not exist any constant relation. On the contrary, when the dropsy is very extensive, eclampsia is very rare, for the blood gets rid of a part of the retained urea by the serous secretions, and the uræmic intoxication may thus be kept off."

It is well known to obstetricians that the eclampsia of pregnant and parturient women is accompanied with great danger to the life of the fœtus. Kiwisch attributes the death of the fœtus under these circumstances to the stoppage of the circulation in the maternal vessels of the placenta during the fits. Our author refers it chiefly to the presence of carbonate of ammonia in the fœtal blood. In proof of this he asserts that

"If, after numerous uræmic convulsive fits, the child is born still alive, a large quantity of urea is found in the blood taken from the umbilical cord; but if it is born dead, we can, immediately after the birth, demonstrate the presence of carbonate of ammonia in the fœtal blood."

In cases of eclampsia during delivery the mortality of the children amounts to 45 per cent. Where the convulsions occur after delivery, the mortality of the children born prematurely is 64 per cent.; and of those born at the full time 40 per cent.

Chapters five and six are taken up with the consideration of the etiology and pathological anatomy of uræmic eclampsia. In chapter seventh many important facts and arguments are brought forward to prove that a very intimate connection exists between eclampsia and uræmia. The differential diagnosis of the disease in question and other affections of the motor system of nerves is considered at length in chapter eighth. Chapters nine and ten treat of the prognosis and treatment of eclampsia and Bright's disease. Upon all these topics Dr. Braun has collected much valuable information which will fully repay an attentive perusal. Upon the whole this work is the most complete and erudite essay upon the subject of which it treats that we are acquainted with, and we only regret that our limited space prevents us from more fully laying before our readers the peculiar views of its author.

Of the manner in which the translation has been accomplished we cannot speak favorably. Though it appears to be a faithful rendering, it can by no means lay claim to elegance. The language is in many instances vague, obscure, and not wholly free from the peculiar idiomatic expressions of the German tongue; the sentences are often involved and clumsy, and occasionally the grammar halts.

